# Health-Related Quality of Life and Life Satisfaction in a Greek Refugee Accommodation Center

**DOI:** 10.7759/cureus.42005

**Published:** 2023-07-17

**Authors:** Thomas D Plakas, Vassilis H Aletras, Eirini I Voutsa, Dimitris Niakas

**Affiliations:** 1 School of Social Sciences, Hellenic Open University, Patras, GRC; 2 Department of Business Administration, University of Macedonia, Thessaloniki, GRC; 3 School of Informatics, Aristotle University of Thessaloniki, Thessaloniki, GRC; 4 Department of Health Economics, National and Kapodistrian University of Athens, Athens, GRC

**Keywords:** asylum seekers, refugees, life satisfaction, health-related quality of life, quality of life

## Abstract

Introduction

In recent years, a large number of refugees have crossed the Greek borders. The aim of this study was to estimate the health-related quality of life (HRQoL) and life satisfaction (LS) of refugees and asylum seekers residing in the Vagiochori Accommodation Center.

Methods

The Short Form-36 (SF-36) survey tool and the Satisfaction With Life Scale (SWLS) were employed for the analysis. The sample consists of 144 individuals with an average age of 39.4 years, most of which are Afghans, married, and have a secondary education level. Non-parametric tests examined the association of respondents’ demographics and health-related and residence-related characteristics with the physical component scale (PCS) and mental component scale (MCS) of SF-36 and SWLS. Regression analysis was used to examine the effect of these variables on dependent scales.

Results

HRQoL and LS of the participants were poor (median scores: PCS = 44.91, MCS = 42.05, and SWLS = 12.00). Age, gender, education, marital status, and specific health-related and residence-related characteristics were associated with HRQoL (p < 0.05). Marital status and specific health-related and residence-related characteristics were associated with SWLS.

Conclusion

In summary, social support, legal counseling, and a better understanding of refugees’ concerns are required to improve refugees’ and asylum seekers’ HRQoL and LS.

## Introduction

The European immigration and refugee crisis of 2015 resulted from the arrival of more than one million people in Southern Europe and especially in Greece and Italy. People from Syria, Afghanistan, and in general from Asia and Africa fled their homelands, looking for a better life in Europe. During 2016-2021, more than 350,000 refugees crossed the Greek borders, forcing respective authorities to act accordingly [1].

The World Health Organization (WHO) Regional Office for Europe suggests that refugees and migrants may be healthy before fleeing their countries, but have an increased risk of getting ill while staying in host areas or during their movement from one location to another. Poor living conditions, lifestyle changes, and uncertainty about the time required to reach the destination make them vulnerable to communicable and non-communicable diseases [2]. Cardiovascular events, dermatological or respiratory illnesses, and gynecological or obstetric complications in combination with mental health problems like depression, anxiety disorder, or post-traumatic disorders affect their health status, having also a negative effect on life satisfaction (LS). Medical actors have to face the burden of the aforementioned diseases that are mainly related to physical, mental, and social problems. Furthermore, given the significant barriers to healthcare access that newly arrived refugees face (i.e., legal issues, bureaucratic difficulties, and lack of communication) [3], the evaluation of health-related quality of life (HRQoL) and LS seems to be of profound importance for designing new policies and reassessing existing ones.

HRQoL has been variously approached. One definition focuses on individual functionality according to the person’s well-being, taking into account physical, mental, and social health aspects. Others describe HRQoL by focusing on the quality of life aspects that are related to the presence or absence of diseases. Lastly, the notion has been used to assign values to different health states, by calculating utilities to be used in the computation of quality-adjusted life years [4].

LS relates to how much a person likes their life and is perceived as a cognitive and overall assessment of the quality of life. It is thought to be affected by factors, like gender, age, personality, health status, job, family, lifestyle, and leisure. Some authors think of it as a complex function of several aspects of LS, with different individuals assessing each domain differently. It has also been seen as a result of personality and other stable characteristics [5,6].

HRQoL of refugees has been estimated using different survey tools. Studies using the World Health Organization Quality of Life, Brief Version (WHOQOL-BREF) questionnaire in Kurdistan and Southwest Nigeria revealed respondents’ higher physical health in comparison with their mental health [7,8]. Moreover, a cross-sectional study of 1,215 Syrian refugees in Sweden employing the EuroQol 5 Dimension 5 Level (EuroQol-5D-5L) questionnaire reported depression as the major HRQoL problem [9]. Moreover, age, gender, duration of the asylum procedure, educational level, type of accommodation, and social support had an impact on HRQoL [9-13].

LS levels of migrant people, having already applied for asylum or having appealed against a negative decision, are lower than the respective levels of recognized refugees. Additionally, residing in private facilities results, on average, in higher LS in comparison with the alternative of refugee accommodation centers [14]. Determinants like age, gender, educational level, social support, and integration affect LS [14-17].

This study aims at measuring the HRQoL and LS of refugees and asylum seekers residing in the Vagiochori Accommodation Center (Thessaloniki, Greece). It has a capacity of 792 persons, while 224 adults and 267 children were present at the camp, during the study period. We examined the impact of several demographics and health-related and residence-related variables on HRQoL and LS. The present study seems to be the second in Greece that assesses the HRQoL of refugees and asylum seekers residing in accommodation centers, following work published in 2022 [13]. Regarding LS, our study provides empirical evidence for the first time.

## Materials and methods

Participants and data collection

During November 2021, with the aid of Arabic and Farsi translators, who were independent of the research group, adult residents of the Vagiochori Accommodation Center were informed about the study goals, voluntary nature of participation, assurance of anonymity, and absence of rewards for questionnaire completion. This information was also provided in the questionnaire itself. Informed consent was obtained from study participants. Residents who were willing to complete the questionnaire did not have any time restrictions. In any case, 15 minutes were considered adequate for the task. A member of the research group and one Arabic and one Farsi translators were available to explain any arising questions. The research was reviewed and approved by the managerial staff of the accommodation center, operating under the guidelines of the Reception and Identification Service of the Greek Ministry of Migration and Asylum.

Study tools

The questionnaire of the study consisted of three main parts. The first one had questions about the independent variables of the research, while the second and the third part contained Short Form-36 (SF-36) tools and Satisfaction With Life Scale (SWLS), respectively. Regarding the independent variables of the study, except demographic data (i.e., age, gender, country of origin, marital status, and educational level), residence-related information (i.e., coverage of needs by non-governmental organizations (NGOs), duration of stay in Greece, and belief about arrival in destination country soon/not soon) and health-related information (i.e., suffering from a serious health problem and medication taking) were collected. SF-36 is a questionnaire suitable for surveys of the general population and healthy people [18]. It contains 36 items, with 35 of them representing eight scales (physical activity (PA), physical role (PR), bodily pain (BP), general health (GH), vitality (VI), social activity (SA), emotional role (ER), and mental health (MH)) and two component scales (physical component scale (PCS) and mental component scale (MCS)). The 36th question is not related to any scale, displaying merely health levels change during the last year [19]. SWLS is a commonly used five-item instrument employing a seven-point Likert scale, which is suitable for surveys concerning individuals in various age groups and different communities [20]. Its scoring ranges from 5 to 35. Scores between 5 and 9 imply very dissatisfied persons, while scores above 31 indicate very satisfied respondents. The neutral point of the scale is 20 [21].

As the residents in Vagiochori were native Farsi and Arabic speakers, the first and the third part of the study were translated into the Farsi and Arabic languages. SWLS was forward-backward translated by independent translators and there were no discrepancies in the translations or issues to be discussed by the research group. To further assess validity, it was presented to 10 refugees and asylum seekers with different characteristics, and the relevance and clarity of the five items were documented. Cronbach's alpha coefficient was 0.86, indicating very good internal consistency reliability. The Farsi edition of SF-36 was available and already validated by Montazeri et al. (2005) [22]. The Arabic edition of SF-36 was available and already validated by Sabbah et al. (2003) [23].

Statistical analysis

Responses to the questionnaire were descriptively analyzed. PCS, MCS, and SWLS scores’ normality assumptions were examined with the use of the Kolmogorov-Smirnov test. Spearman correlation along with Mann-Whitney U and Kruskal-Wallis tests were employed to examine the association of the demographic, residence, and health-related characteristics with the three quality of life and satisfaction scales. For each category of the categorical/ordinal variables, separate scores of the three dependent scales were presented. To compare the variability of the three scale scores among different groups of categorical/ordinal characteristics, a nonparametric test of equality of variances was employed [24]. For equal distributions of the scale scores between two or more groups of a categorical/ordinal variable, the median score was preferred, rather than the mean rank, to compare the data. Otherwise, the mean rank was the appropriate indicator.

Three multiple linear regressions assessed the effect of the independent variables on PCS, MCS, and SWLS scores. The selection of the independent variables was based on Spearman correlation, Mann-Whitney U test, and Kruskal-Wallis test p-values between those characteristics and the dependent scales. A p-value cutoff < 0.1 was applied to the analysis. Therefore, the variable “suffering from a serious health problem” was eligible for all the models of the analysis, while “coverage of needs by NGOs” was eligible for the MCS and SWLS models. Regression residuals’ distributions were visually examined with normal P-P plots, which indicated no significant departures from normality. Dummy variables were used to capture the aforementioned categorical/ordinal data. In cases of variables with more than two categories, one was excluded from the regressions and acted as the reference category. Statistical significance was set at 0.05. Statistical analyses were conducted using SPSS version 26.0 (IBM Corp., Armonk, NY).

## Results

The survey questionnaire was distributed to 224 adult residents of the Vagiochori Accommodation Center. A total of 144 persons agreed to participate and completed it. Therefore, the response rate of the study is 64.28%.

Demographics, residence-related, and health-related results

Table 1 presents the demographic, health-related, and residence-related data of the survey. Respondents had a median age of 37.50 years. The majority of them were male (56.94%), married with their spouses also living in the camp (75.70%), and came from Afghanistan (83.33%). Most of them had completed secondary education (51.39%), did not report a serious health problem (58.33%), and were not taking any medication despite having access to health care and medicines (71.53%). The larger part of them mentioned that NGOs were not covering their needs (in passing, digital health is in fact not available to them) (60.42%), expected to reach their destination soon (56.25%), and were hosted in Greece for more than one year (71.53%).

**Table 1:** Characteristics of study participants (n = 144) IR: interquartile range.

Median, mean, IR		Frequency (%)			Frequency (%)		
Demographic data		Demographic data			Residence-related data		
Age		Marital status			Coverage of needs by NGOs		
37.50, 39.41, 22.00		Single		23 (15.97)	Yes		57 (39.58)
Frequency (%)		Married		109 (75.70)	No		87 (60.42)
Country of origin		Divorced/widowed		12 (8.33)	Duration of stay in Greece		
Afghanistan	120 (83.33)	Education			<12 months		41 (28.47)
Syria	10 (6.95)	Primary	53 (36.80)		>12 months		103 (71.53)
Iran	5 (3.47)	Secondary	74 (51.39)		Expectations about arrival in the destination country		
Kuwait	4 (2.78)	Tertiary	17 (11.81)		Soon	81 (56.25)	
Iraq	3 (2.08)	Health-related data			Not soon	63 (43.75)	
Palestine	2 (1.39)	Medication taking					
Gender		Yes	41 (28.47)				
Male	82 (56.94)	No	103 (71.53)				
Female	62 (43.06)	Serious health problem					
		Yes	60 (41.67)				
		No	84 (58.33)				

HRQoL and LS measurements

PCS, MCS, and SWLS scores were not normally distributed (Kolmogorov-Smirnov test p-values were 0.032, 0.001, and 0.000, respectively). Table 2 shows HRQoL elicited with SF-36 and LS according to SWLS. Regarding HRQoL, ER had the highest median score (100.00), while GH and VI presented the lowest median scores (51.00 and 55.00, respectively). PCS, MCS, and SWLS medians were 44.91, 42.05, and 12.00, respectively.

**Table 2:** Health-related quality of life (SF-36) and life satisfaction (SWLS) SF-36: Short Form-36; IR: interquartile range.

	Median	Mean	IR	Minimum	Maximum
Physical activity (PA)	80.00	72.60	48.75	0.00	100.00
Bodily pain (BP)	60.50	57.71	65.00	0.00	100.00
Physical role (PR)	75.00	60.07	93.75	0.00	100.00
General health (GH)	51.00	51.11	39.50	0.00	100.00
Vitality (VI)	55.00	56.01	35.00	0.00	100.00
Social activity (SA)	62.50	59.64	50.00	0.00	100.00
Emotional role (ER)	100.00	59.72	100.00	0.00	100.00
Mental health (MH)	60.00	58.14	32.00	0.00	100.00
Physical component scale (PCS)	44.91	43.90	17.75	20.82	62.70
Mental component scale (MCS)	42.05	42.08	19.47	11.66	63.91
Satisfaction with life scale (SWLS)	12.00	13.64	12.00	5.00	35.00

Table 3 shows scale scores across different sub-groups of participants, taking into consideration their characteristics. For the continuous variable “age”, Spearman correlation was used. PCS was significantly associated with age (p = 0.000), education (p = 0.000), marital status (p = 0.000), medication taking (p = 0.000), suffering from a serious health problem (p = 0.000), duration of stay in Greece (p = 0.011), and expectation about arrival in destination country soon/not soon (p = 0.000). Furthermore, MCS was significantly associated with age (p = 0.015), gender (p = 0.029), education (p = 0.004), marital status (p = 0.007), medication taking (p = 0.000), suffering from a serious health problem (p = 0.000), duration of stay in Greece (p = 0.000), and expectation about arrival in destination country soon/not soon (p = 0.003). Summarily, younger respondents presented higher HRQoL and female participants’ MCS scores were higher than those of male participants. SWLS was significantly associated with marital status (p = 0.028), medication taking (p = 0.012), coverage of needs by NGOs (p = 0.000), and expectation about arrival in the destination country soon/not soon (p = 0.000). Participants who believed that they would arrive in their destination country soon presented higher SWLS scores.

**Table 3:** Physical component scale (PCS), mental component scale (MCS), and Satisfaction With Life Scale (SWLS) scores by respondents’ characteristics ^a ^Spearman correlation results for continuous variable “Age”, α = 0.05; ^b ^Mann-Whitney U test, α = 0.05. For equal distributions of the dependent scales’ scores between different groups of the categorical/ordinal variables, the median score was preferred against the mean rank to compare the data. For non-equal distributions, mean rank was more suitable. ^c ^Kruskal-Wallis test, α = 0.05. SRCC: Spearman's rank correlation coefficient; NGOs: non-governmental organizations; MIN: minimum; MAX: maximum; N/A: not applicable.

	PCS					MCS					SWLS				
Age	SRCC			P-value		SRCC			P-value		SRCC			P-value	
	-0.516^a^			0.000^a^		-0.202^a^			0.015^a^		-0.091^a^			0.279^a^	
	Median	Mean rank	Min	Max	P-value	Median	Mean rank	Min	Max	P-value	Median	Mean rank	Min	Max	P-value
Gender					0.173^b^					0.029^b^					0.876^b^
Male	43.58	N/A	20.82	60.71		36.98	N/A	11.66	63.91		12.00	N/A	5	35	
Female	47.98	N/A	21.27	62.70		47.84	N/A	19.00	61.14		11.50	N/A	5	35	
Education					0.000^c^					0.004^c^					0.727^c^
Primary	N/A	54.98	21.27	62.70		N/A	58.47	11.66	59.03		11.00	N/A	5	35	
Secondary	N/A	80.76	23.76	60.71		N/A	78.18	19.73	63.91		12.50	N/A	5	30	
Tertiary	N/A	91.18	20.82	59.60		N/A	91.53	19.00	58.11		14.00	N/A	5	23	
Marital status					0.000^c^					0.007^c^					0.028^c^
Single	55.60	N/A	39.54	62.70		51.59	N/A	24.02	61.14		17.00	N/A	5	30	
Married	43.28	N/A	20.82	60.71		37.17	N/A	11.66	63.91		11.00	N/A	5	35	
Divorced/widowed	39.93	N/A	26.87	56.62		45.18	N/A	28.73	60.03		16.00	N/A	5	30	
Medication taking					0.000^b^					0.000^b^					0.012^b^
Yes	N/A	37.56	20.82	54.54		N/A	44.49	21.67	58.69		10.00	N/A	5	31	
No	N/A	86.41	21.27	62.70		N/A	83.65	11.66	63.91		13.00	N/A	5	35	
Serious health problem					0.000^b^					0.000^b^					0.065^b^
Yes	33.52	N/A	20.82	60.40		38.04	N/A	11.66	63.91		11.00	N/A	5	31	
No	49.76	N/A	21.27	62.70		50.60	N/A	20.10	61.14		13.00	N/A	5	35	
Coverage of needs by NGOs					0.390^b^					0.095^b^					0.000^b^
Yes	46.96	N/A	20.82	62.70		N/A	79.67	19.00	61.14		17.00	N/A	5	35	
No	44.50	N/A	21.27	60.71		N/A	67.80	11.66	63.91		10.00	N/A	5	26	
Duration of stay in Greece					0.011^b^					0.000^b^					0.389^b^
<12 months	49.48	N/A	26.25	60.48		51.33	N/A	23.55	61.14		N/A	67.77	5	25	
>12 months	43.06	N/A	20.82	62.70		37.17	N/A	11.66	63.91		N/A	74.38	5	35	
Expectations about arrival in the destination country					0.000^b^					0.003^b^					0.000^b^
Soon	49.30	N/A	20.82	62.70		47.31	N/A	11.66	63.91		15.00	N/A	5	35	
Not soon	40.14	N/A	21.27	60.71		36.87	N/A	19.73	60.89		10.00	N/A	5	31	

The variables “education” and “marital status” have three groups (categories) each. Table 4 shows associations between each possible pair of the groups with PCS, MCS, and SWLS scores. The non-significant association between education and SWLS score is not reported in the aforementioned table.

**Table 4:** Differences between groups of the independent variables on the physical component scale (PCS), mental component scale (MCS), and Satisfaction With Life Scale (SWLS) ^a ^Kruskal-Wallis test, α = 0.05.

	PCS	MCS	SWLS
	P-value	P-value	P-value
Education			
Primary - secondary	0.002^a^	0.026^a^	Not applicable
Primary - tertiary	0.006^a^	0.013^a^	Not applicable
Secondary - tertiary	1.000^a^	0.702^a^	Not applicable
Marital status			
Single - married	0.000^a^	0.007^a^	0.029^a^
Single - divorced/widowed	0.001^a^	1.000^a^	0.070^a^
Married - divorced/widowed	1.000^a^	0.623^a^	0.890^a^

Effect of independent variables on PCS, MCS, and SWLS scores

Multiple linear regression results are presented in Table 5. A total of 48.7% of PCS variation is explained by the first model. Growing older and suffering from a serious health problem had a negative impact on PCS (p = 0.002 and p = 0.000, respectively). Moreover, 34.0% of MCS variation is explained by the second model. Being accommodated in Greece for more than one year and suffering from a serious health problem had a negative effect on MCS (p = 0.042 and p = 0.000, respectively). The effect of the variable “suffering from a serious health problem” on HRQoL was strong. Respondents with a serious health problem presented, on average, 8.656% lower PCS than healthy people. Their MCS scores were, on average, 10.029% lower as compared to healthy participants. Last but not least, 24.1% of SWLS variation is explained by the third model. Coverage of needs by NGOs had a positive impact on SWLS (p = 0.000), while medication taking had a negative effect on it (p = 0.031). Participants who believed that NGOs covered their needs had, on average, 6.736% higher SWLS scores in comparison with unsatisfied persons. Participants who were taking medication had, on average, 3.611% lower SWLS scores than persons without this characteristic.

**Table 5:** Multiple linear regressions of physical component scale (PCS), mental component scale (MCS), and Satisfaction With Life Scale (SWLS) scores

	PCS			MCS			SWLS		
	Coefficient	Standard error	P-value	Coefficient	Standard error	P-value	Coefficient	Standard error	P-value
Constant	53.134	3.156	0.000	43.746	3.972	0.000	10.282	1.102	0.000
Age	-0.175	0.057	0.002	0.128	0.074	0.088	Not applicable		
Gender (male)	Not applicable			-2.613	1.737	0.135	Not applicable		
Coverage of needs by NGOs (Yes)	Not applicable			-0.184	1.831	0.920	6.736	1.203	0.000
Duration of stay in Greece (>12 months)	-0.920	1.531	0.549	-3.958	1.929	0.042	Not applicable		
Expectation about arrival in the destination country (soon)	1.768	1.416	0.214	1.405	1.923	0.466	1.395	1.203	0.248
Medication taking (Yes)	-1.647	2.078	0.429	-2.529	2.627	0.337	-3.611	1.653	0.031
Serious health problem (Yes)	-8.656	1.875	0.000	-10.029	2.375	0.000	2.025	1.525	0.186
Secondary education (reference category: primary education)	0.805	1.524	0.598	1.292	1.915	0.501	Not applicable		
Tertiary education (reference category: primary education)	3.026	2.373	0.204	3.551	2.986	0.236	Not applicable		
Single (reference category: married, divorced/widowed)	4.061	2.106	0.056	4.460	2.721	0.103	0.567	1.533	0.712
Adjusted R^2^	0.487			0.340			0.241		

## Discussion

This study is the second that estimates HRQoL among refugees and asylum seekers residing in accommodation centers in Greece, following the findings of prior research [13]. Additionally, it is the first research providing evidence on LS. HRQoL was poor, with PCS scores being higher than the respective MCS. These conclusions are confirmed by three related studies [7,8,13]. LS levels were low as well, with 75% of the participants scoring below the neutral point of SWLS. A study of Bosnian refugees arriving in Norway during the war in former Yugoslavia, which also used Diener’s scale, reported similar results [15].

Age is a common factor affecting the HRQoL of refugees and migrants, with lower HRQoL documented for elder people [10,12]; younger participants in our survey presented significantly higher PCS and MCS scores than elder ones. In fact, it could be that the former experiences lower morbidity than the latter due to their ability to better adapt to new environmental conditions. Female study participants had better mental health than male respondents. This information is not in accordance with a previous European study [16]. However, as has been noted elsewhere, social support plays an important role in improving women’s HRQoL [10]. Moreover, it has been shown that social support, cohabitation, and age differences may have an impact on the HRQoL of male/female refugees, with more future research required to gain a better understanding [9]. Lastly, the female beneficiaries of Vagiochori accommodation receive a variety of integration and social support services (i.e., a “female-friendly space project” is in effect) from international actors and NGOs [25].

Our study suggests that respondents with serious health problems have lower HRQoL as compared to healthy participants. In addition, the variable “medication taking” was significantly associated with all the scales of the survey (lower scores for people taking medication). Refugees desire to improve their quality of life, while different barriers arise, impacting negatively on their mental health [26], HRQoL, and LS [16].

Coverage of needs by NGOs was positively associated with LS. Beneficiaries who mentioned that NGOs (specializing in educational, recreational, and protection issues) met their expectations presented significantly higher LS scores than unsatisfied respondents. A Spanish study aiming at analyzing the influence of the sense of community on immigrants suggested that becoming socially integrated had a direct relationship with LS. Decreased LS has been explained by different migration-related factors, like the lack of resources to cover basic needs. When the sense of community was higher, the integration of migrants was smoother and LS was similar to that of the native population [17].

Respondents’ expectations about short arrival in destination countries had a positive effect on PCS, MCS, and SWLS. Additionally, PCS and MCS were affected by the duration of participants’ stay in Greece, with longer stays being responsible for lower scores. A study using WHOQOL-BREF reported that a long asylum procedure had a negative impact on the overall quality of life of Iraqi asylum seekers in the Netherlands [11]. Moreover, a German survey of refugees arriving in the country between 2013 and 2016 showed that an uncertain legal status was related to reduced LS [14]. The conclusions of these surveys are in accordance with our findings. In fact, migrant populations with uncertain legal status in Greece do not receive travel documents that allow them to visit other European countries [27]. This characteristic may have affected participants’ HRQoL and LS in our study.

We have also found that respondents with tertiary and secondary education presented higher HRQoL in comparison with those with primary education. There are similar studies that showed a lack of association between education and HRQoL [9,10]. Another study suggested the existence of an association of the MH scale with education [12]. It has also been concluded that well-educated persons have better health outcomes [28]. Lastly, it has been argued that education cannot be considered a socioeconomic marker in refugee populations. Other factors before (i.e., traumatic experiences) and after migration (i.e., social support) may override the potential effect of the variable on health status [9].

PCS scores of single participants were significantly higher than scores of married and divorced/widowed persons. Their MCS was significantly higher only in comparison with married participants. Research has documented similar results regarding the HRQoL of refugees in Germany. Single persons had the highest PCS and MCS scores, whereas divorced/widowed individuals had the lowest ones [29]. Lastly, single respondents were more satisfied than married ones. Regarding family reunification, it has been shown that refugees and asylum seekers who aimed at reuniting with a spouse or with underage children were less satisfied and more distressed [14]. This suggestion provides a good explanation for our findings, taking into account the existing challenges [30].

Although our study found useful evidence on HRQoL and LS, it still has some limitations. The sample was collected only from one accommodation center. Therefore, findings cannot be considered representative of the refugees/asylum seekers residing in Greece. The median age of our sample was 37.50 years whereas, generally, it is the older populations that are associated with significant reductions in quality of life. Moreover, it is not possible to investigate the potential impact of different types of accommodation facilities on HRQoL and LS. Other studies showed that residing in an urban environment/private housing is related to higher HRQoL and LS compared to staying in refugee accommodation centers [13,14]. Finally, our dataset does not allow us to analyze the effect of “country of origin” on the dependent variables of the study. Following the strategy of the Reception and Identification Service of the Greek Ministry of Migration and Asylum about accommodation centers with homogenous populations, 120 participants were from Afghanistan, while only 24 participants were coming from five different countries. According to previous surveys, nationality affects HRQoL and LS [10,14].

## Conclusions

Our study provides empirical evidence on the HRQoL of refugees and asylum seekers in Greek accommodation centers. Furthermore, it is the first study focusing on the LS of this population. The respondents in fact presented poor HRQoL and LS. Policy initiatives should therefore be strengthened to improve their health and life satisfaction. Social integration and engagement of refugees and asylum seekers in local activities in preparation for their life to come in their destination countries might keep them busy and productive and positively distracted from stress and depression. They can use their time in refugee camps to strategize their lives ahead realistically, begin to accept their new life journey albeit the uncertainty, and focus on developing themselves and improving their chances of future integration. This involves inter alia learning the language, the social system, and potential job and other opportunities in the destination country. Access to health care, legal counseling, provision of social support/integration services, and a better understanding of refugees’ concerns (i.e., coverage of basic needs, and reunification issues) are of utmost importance for achieving higher HRQoL and LS. According to our study results, decision-makers should also consider gender, age, education, and other differences among refugees during policy formation and implementation. Along with official government measures, NGOs should be further empowered to fully cover the needs of these populations. Barriers to their journey to the destination countries should be addressed so that the relative uncertainties are minimized and the duration is shortened.

Finally, for policymakers to be able to effectively motivate refugees and asylum seekers to become active members of society, future studies have to assess the relative impact of integration support policies (e.g., employability support, integration courses regarding language learning and life skills, and organization of activities to reinforce the social interaction between the local and the hosted communities) on HRQoL and LS.
